# Stability of Ceftiofur Sodium and Cefquinome Sulphate in Intravenous Solutions

**DOI:** 10.1155/2014/583461

**Published:** 2014-06-03

**Authors:** Agnieszka Dołhań, Anna Jelińska, Marcelina Bębenek

**Affiliations:** Department of Pharmaceutical Chemistry, Faculty of Pharmacy, Poznan University of Medical Sciences, Grunwaldzka 6, 60-780 Poznań, Poland

## Abstract

Stability of ceftiofur sodium and cefquinome sulphate in intravenous solutions was studied. Chromatographic separation and quantitative determination were performed by using a high-performance liquid chromatography with UV-DAD detection. During the stability study, poly(vinylchloride) minibags were filled with a solution containing 5 mg of ceftiofur sodium or cefquinome sulphate and diluted to 0.2 mg/mL with suitable intravenous solution depending on the test conditions. The solutions for the study were protected from light and stored at room temperature (22°C), refrigerated (6°C), frozen (−20°C) for 30 days, and then thawed at room temperature. A comparison of results obtained at 22°C and 6°C for the same intravenous solutions showed that temperature as well as components of solutions and their concentration had an influence on the stability of ceftiofur sodium and cefquinome sulphate. It was found that ceftiofur sodium and cefquinome sulphate dissolved in intravenous solutions used in this study may be stored at room temperature and at 6°C for up to 48 h.

## 1. Introduction

Cephalosporins, a group of *β*-lactam antibiotics, have been used in the treatment of various types of infections since 1954 [1]. They are characterized by a broad spectrum of antimicrobial activity and low toxicity. The mechanism of action of the cephalosporins involves inhibiting the synthesis of bacterial cell wall [2]. In a cephalosporin molecule, the *β*-lactam moiety is essential for antibacterial activity, but it is also very vulnerable to degradation. Products of cephalosporin degradation do not show any antimicrobial activity and may demonstrate a variety of unwanted side effects.

Ceftiofur sodium is a third-generation cephalosporin antibiotic used in veterinary medicine, approved by the Food and Drug Administration (FDA) for intramuscular injection in the treatment of certain respiratory diseases in beef cattle, dairy cattle, day-old chicken, and swine [3–5]. It is also applied to treat respiratory diseases in horses and ruminants [6]. Ceftiofur sodium is active against* Actinobacillus pleuropneumoniae*,* Escherichia coli*,* Haemophilus parasuis*,* Haemophilus somnus*,* Pasteurella haemolytica*,* Pasteurella multocida*, and* Streptococcus suis* [7–10]. Its broad spectrum of activity is attributable in part to its resistance to inactivation by bacterial *β*-lactamase due to the presence of a large iminomethoxy side chain [11]. The thioester bond of ceftiofur is rapidly cleaved to give desfuroylceftiofur which is further metabolized to a disulfide dimer and various desfuroylceftiofur-protein and amino acid conjugates [12, 13].

Cefquinome sulphate is a veterinary, parenteral, and fourth-generation cephalosporin. Its antimicrobial potency and extensive antibacterial spectrum result from the introduction of a methoxyimino-aminothiazolyl moiety into the acyl side chain. This change made it resistant to inactivation by *β*-lactamases [14–16].

Fourth-generation cephalosporins have a broad spectrum of antibacterial activity against Gram-positive and Gram-negative bacteria, including* Pseudomonas aeruginosa* and Enterobacteriaceae [17–19]. Those compounds are also easily transported across the blood-brain barrier [20–27]. They are used to treat infections of the urinary tract, lungs, skin, and soft tissues as well as in postoperative prophylaxis [20, 28].

The cephalosporins are prone to degradation in aqueous solutions [29–32] and in the solid state [33–37]. Since ceftiofur sodium and cefquinome sulfate are administrated parenterally, it is essential to evaluate the influence of intravenous solutions used to dilute the drugs on their stability.

## 2. Experimental

Ceftiofur sodium was obtained from MOLEKULA (Shaftesbury, United Kingdom) and cefquinome sulphate from BePharm Ltd. (China).

Water for injections was obtained from Polpharma SA (Poland). Sodium chloride (9 *μ*g/mL), glucose (0.05 mg/mL, 0.1 mg/mL, and 0.2 mg/mL), multielectrolytic isotonic fluid, pediatric fluid, and Ringer's solution were products of Baxter Manufacturing Sp. Z o. o. (Poland). Solutio Ringeri Lactate, mixture of 9 *μ*g/mL sodium chloride, and 0.5 mg/mL glucose (1 : 1 *V*/*V*, 1 : 2 *V*/*V*) were products of Fresenius Kabi, Italy.

All other chemicals and solvents were obtained from Merck KGaA (Germany) and were of analytical or high-performance liquid chromatographic grade. High-quality pure water was prepared by using an Exil SA 67120 Millipore purification system (Millipore, Molsheim, France).

Chromatographic separation and quantitative determination were performed by using a high-performance chromatograph Shimadzu LC-20AT (ceftiofur sodium) and Shimadzu LC-6A (cefquinome sulphate). As stationary phase, LiChroCART RP-18e column (5 *μ*m, 250 mm × 4.6 mm) (Merck, Darmstadt, Germany) and LiChroCART RP-18 (5 *μ*m, 125 mm × 4 mm) (Merck, Darmstadt, Germany) were used for ceftiofur sodium and cefquinome sulphate, respectively. The mobile phase consisted of 22 volumes of acetonitrile and 78 volumes of phosphate buffer (0.02 M, pH = 6.0) for ceftiofur sodium and 10 volumes of acetonitrile and 90 volumes of phosphate buffer (0.02 M, pH = 7.0) for cefquinome sulphate. The flow rate of the mobile phase was 1.2 mL/min for ceftiofur sodium and 1.0 mL/min for cefquinome sulphate. The wavelength of the UV-DAD detector was set at 292 nm and 268 nm for ceftiofur sodium and cefquinome sulphate, respectively. The HPLC method for ceftiofur sodium determination was developed by Souza et al. [38] and modified and validated in the Department of Pharmaceutical Chemistry, Poznan University of Medical Sciences [39].

## 3. Sample Preparation

During the stability study, poly(vinylchloride) minibags were filled with a solution containing 5 mg of ceftiofur sodium or cefquinome sulphate and diluted to 0.2 mg/mL with water for injections, sodium chloride (9 *μ*g/mL), glucose (0.05 mg/mL, 0.1 mg/mL, and 0.2 mg/mL), multielectrolytic isotonic fluid, pediatric fluid, Ringer's solution, Solutio Ringeri Lactate, and mixture of 9 *μ*g/mL sodium chloride and 0.05 mg/mL glucose (1 : 1 *V*/*V*, 1 : 2 *V*/*V*), depending on the test conditions. The solutions for the study were protected from light and stored at room temperature (22°C), refrigerated (6°C) or frozen (−20°C) for 30 days, and then thawed at room temperature.

At specified time intervals samples were collected and 50 *μ*L of the solutions was injected onto the column.

## 4. Results and Discussion

**Figure 1:**
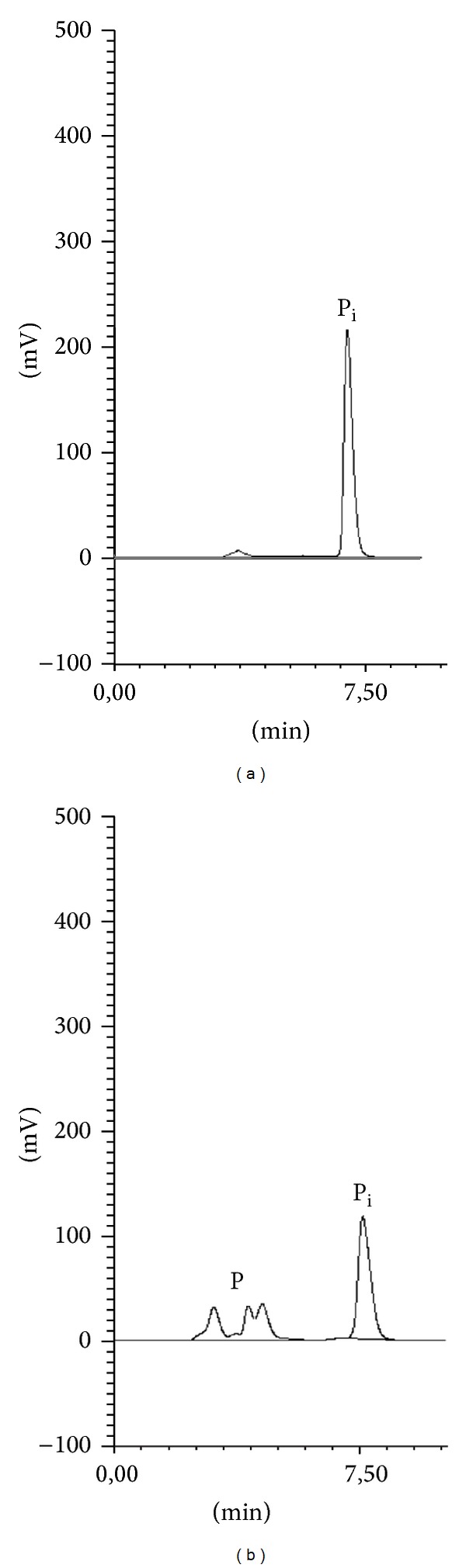
HPLC chromatogram of ceftiofur sodium after incubation (a) 0 h and (b) 23 days in 0.2 mg/mL glucose in 22°C (P_i_: ceftiofur sodium, P: degradation products).

**Figure 2:**
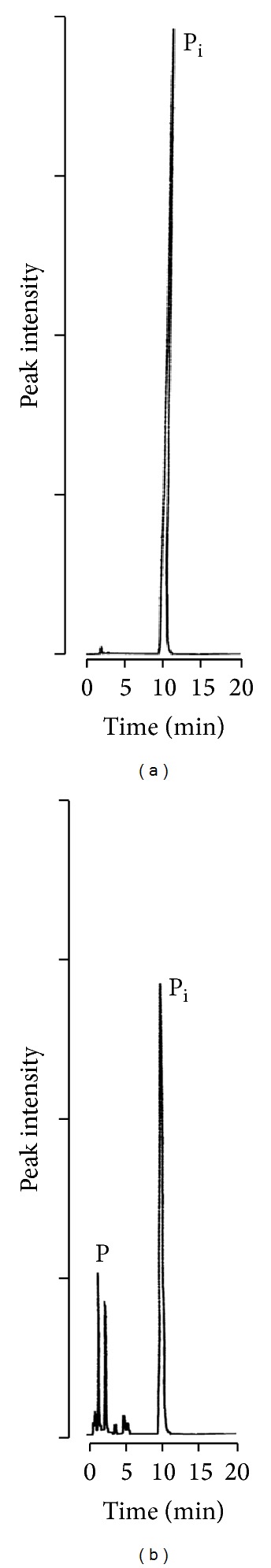
HPLC chromatogram of cefquinome sulphate after incubation (a) 0 h and (b) 6 days in 0.2 mg/mL glucose in 22°C (P_i_: cefquinome sulphate, P: degradation products).

Although the HPLC method with UV detection was previously found suitable for the determination of ceftiofur sodium [39] and cefquinome sulphate [40] under the stress conditions of hydrolysis (acid and base), oxidation, photolysis, and thermal degradation, its selectivity in the presence of degradation products was confirmed (Figures 1 and 2).

Solutions of ceftiofur sodium and cefquinome sulphate were defined as stable when the substrate loss was not greater than 10% relative to the initial value. When degradation exceeded 10%, the observed rate constants were determined (Tables 1, 2, and 3).

Ceftiofur sodium and cefquinome sulphate were degraded according to pseudo-first-order reactions described by the following equation:

(1) $$\begin{array}{c} \ln{\text{P}}_{\text{it}}=\ln{\text{P}}_{\text{i}0}-k_{\text{obs}}\cdot t, \end{array}$$

where P_it_ and P_i0_ are the areas of the peaks of ceftiofur sodium or cefquinome sulphate, at time = 0 and *t*, respectively.

The study showed that at 6°C ceftiofur sodium was stable (substrate loss not greater than 10%) in water for injection, 9 *μ*g/mL sodium chloride, 0.05 mg/mL and 0.1 mg/mL glucose, mixture of 9 *μ*g/mL sodium chloride and 0.05 mg/mL glucose (1 : 1 *V*/*V* and 1 : 2 *V*/*V*), Ringer's solution, Solutio Ringeri Lactate, multielectrolytic isotonic fluid, and pediatric fluid. The degradation of ceftiofur sodium in 0.2 mg/mL glucose exceeded 0.1 mg/mL of the initial concentration. At −20°C, all solutions of ceftiofur sodium were found to be stable.

At 22°C, ceftiofur sodium was the most stable in Ringer's solution and water for injection, with 92.60% and 91.92% of the initial concentration after 23 days of testing, respectively. It was the most unstable in 0.2 mg/mL glucose, with 72.86% of the initial concentration after 23 days. At 22°C in 9 *μ*g/mL sodium chloride, 0.05 mg/mL glucose, mixture of 9 *μ*g/mL sodium chloride and 0.05 mg/mL glucose (1 : 1 *V*/*V*), multielectrolytic isotonic fluid, and pediatric fluid after 2 days of incubation, there were no changes in the concentrations. As a precipitate was formed after 6 days of incubation, the experiment was abandoned.

At 6°C, ceftiofur sodium was the most stable in pediatric fluid (after 28 days with 98.12% of the initial concentration) and the most unstable in 0.2 mg/mL glucose (after 28 days with 89.59% of the initial concentration). After 30 days of incubation at −20°C, ceftiofur sodium was the most stable in pediatric fluid (after 30 days with 98.98% of the initial concentration) and the most unstable in 9 *μ*g/mL sodium chloride (after 30 days with 89.99% of the initial concentration).

The study demonstrated that at 6°C cefquinome sulphate was stable (substrate loss not greater than 10% for 6 days) in water for injection, 9 *μ*g/mL sodium chloride, 0.05 mg/mL and 0.1 mg/mL glucose, mixture of 9 *μ*g/mL sodium chloride and 0.05 mg/mL glucose (1 : 1 *V*/*V*), multielectrolytic isotonic fluid, and pediatric fluid. All the solutions were stable at 6°C and after 30 days of incubation at −20°C.

At 22°C, cefquinome sulphate was the most stable in 9 *μ*g/mL sodium chloride (after 6 days of incubation, no changes in the concentrations were observed) and the most unstable in 0.1 mg/mL glucose (after 6 days with 75.34% of the initial concentration). At 6°C, it was the most stable in multielectrolytic isotonic fluid (after 10 days with 93.29% of the initial concentration) and pediatric fluid (after 9 days with 93.69% of the concentration). At 6°C, it was the most unstable in 0.1 mg/mL glucose (after 9 days with 89.99% of the initial concentration).

At −20°C, cefquinome sulphate was the most stable in a mixture of 9 *μ*g/mL sodium chloride and 0.05 mg/mL glucose (1 : 1 *V*/*V*), Ringer's lactate solution, and pediatric fluid (no changes in the concentrations were observed after 30 days of incubation). It was the most unstable in a mixture of 9 *μ*g/mL sodium chloride and 0.05 mg/mL glucose (2 : 1 *V*/*V*) (after 30 days with 92.89% of the initial concentration).

A comparison of results obtained at 22°C and 6°C for the same intravenous solutions showed that temperature as well as components of solutions and their concentration had an influence on the stability of ceftiofur sodium and cefquinome sulphate.

It was found that ceftiofur sodium and cefquinome sulphate dissolved in intravenous solutions used in those studies may be stored at room temperature and at 6°C for up to 48 h.

## Figures and Tables

**Table 1:** Stability of ceftiofur sodium stored in poly(vinyl chloride) minibags.

22°C			6°C		
Intravenous solution	*t* (day)	*c* (%)	Intravenous solution	*t* (day)	*c* (%)
Water for injection	0	100.00	Water for injection	0	100.00
	2	98.85		3	99.02
	6	97.62		12	97.13
	23	91.92		28	94.23

Glucose 0.1 mg/mL	0	100.00	Glucose 0.1 mg/mL	0	100.00
	2	98.40		3	99.28
	6	95.61		12	97.21
	23	82.87		28	91.35

Glucose 0.2 mg/mL	0	100.00	Glucose 0.2 mg/mL	0	100.00
	2	97.73		3	98.39
	6	92.27		12	94.96
	23	72.86		28	89.59

Mixture of 9 *μ*g/mL sodium chloride and 0.05 mg/mL glucose (1 : 2 *V*/*V*)	0	100.00	Mixture of 9 *μ*g/mL sodium chloride and 0.05 mg/mL glucose (1 : 2 *V*/*V*)	0	100.00
	2	98.14		3	99.32
	6	96.10		12	98.80
	23	87.33		28	97.05

Solutio Ringeri	0	100.00	Solutio Ringeri	0	100.00
	2	98.71		3	99.14
	6	97.78		12	97.76
	23	92.60		28	95.77

Solutio Ringeri Lactate	0	100.00	Solutio Ringeri Lactate	0	100.00
	2	99.18		3	99.46
	6	96.30		12	98.81
	23	87.65		28	97.56

Multielectrolytic isotonic fluid	0	100.00	Multielectrolytic isotonic fluid	0	100.00
	2	100.16		3	99.12
	6	Precipitation		12	98.04
				28	95.11

Pediatric fluid	0	100.00	Pediatric fluid	0	100.00
	2	99.56		3	99.65
	6	Precipitation		12	99.21
				28	98.12

Sodium chloride 9 *μ*g/mL	0	100.00	Sodium chloride 9 *μ*g/mL	0	100.00
	2	100.02		3	99.61
	6	Precipitation		12	99.02
				28	97.88

Glucose 0.05 mg/mL	0	100.00	Glucose 0.05 mg/mL	0	100.00
	2	100.15		3	99.02
	6	Precipitation		12	97.88
				28	94.79

Mixture of 9 *μ*g/mL sodium chloride and 0.05 mg/mL glucose (1 : 1 *V*/*V*)	0	100.00	Mixture of 9 *μ*g/mL sodium chloride and 0.05 mg/mL glucose (1 : 1 *V*/*V*)	0	100.00
	2	99.77		3	99.44
	6	Precipitation		12	98.72
				28	96.15

*c* (%): percent of initial concentration.

**Table 2:** Stability of cefquinome sulphate stored in poly(vinyl chloride) minibags.

22°C			6°C		
Intravenous solution	*t * (day)	*c* (%)	Intravenous solution	*t* (day)	*c* (%)
Water for injection	0	100.00	Water for injection	0	100.00
	1	98.28		1	98.52
	3	96.42		3	96.53
	6	91.93		9	91.92

Sodium chloride 9 *μ*g/mL	0	100.00	Sodium chloride 9 *μ*g/mL	0	100.00
	1	99.50		1	98.24
	3	99.90		3	96.30
	6	99.84		9	91.42

Glucose 0.05 mg/mL	0	100.00	Glucose 0.2 mg/mL	0	100.00
	1	98.20		1	98.12
	3	89.75		3	95.02
	6	78.88		9	90.81

Glucose 0.1 mg/mL	0	100.00	Glucose 0.1 mg/mL	0	100.0
	1	94.73		1	98.53
	3	85.69		3	96.30
	6	75.34		9	89.99

Glucose 0.2 mg/mL	0	100.00	Glucose 200 g/L	0	100.00
	1	96.46		1	98.30
	3	91.14		3	96.15
	6	82.41		9	92.10

Mixture of 9 *μ*g/L sodium chloride and 0.05 mg/mL glucose (1 : 1 *V*/*V*)	0	100.00	Mixture of 9 *μ*g/mL sodium chloride and 0.05 mg/mL glucose (1 : 1 *V*/*V*)	0	100.00
	1	99.48		1	99.72
	3	96.41		3	97.32
	6	91.01		9	96.41

Mixture of 9 *μ*g/mL sodium chloride and 0.05 mg/mL glucose (1 : 2 *V*/*V*)	0	100.00	Mixture of 9 *μ*g/mL sodium chloride and 0.05 mg/mL glucose (1 : 2 *V*/*V*)	0	100.00
	1	96.41		1	99.55
	3	92.06		3	98.38
	6	81.73		9	92.27

Solutio Ringeri	0	100.00	Solutio Ringeri	0	100.00
	1	97.89		1	99.78
	3	92.27		3	97.58
	6	82.86		9	91.99

Solutio Ringeri Lactate	0	100.00	Solutio Ringeri Lactate	0	100.00
	1	97.88		1	98.66
	3	92.70		3	98.44
	6	86.12		9	93.56

Multielectrolytic isotonic fluid	0	100.00	Multielectrolytic isotonic fluid	0	100.00
	1	98.14		1	99.95
	3	95.72		3	98.11
	6	92.13		9	95.13

Pediatric fluid	0	100.00	Pediatric fluid	0	100.00
	1	97.13		1	98.16
	3	93.88		3	96.16
	6	87.81		9	93.69

*c* (%): percent of initial concentration.

**Table 3:** Stability of ceftiofur sodium and cefquinome sulphate stored for 30 days at −20°C in poly(vinyl chloride) minibags.

Solution	Ceftiofur sodium		Cefquinome sulphate	
	*t* (day)	*c* (%)	*t* (day)	*c* (%)
Water for injection	0	100.00	0	100.00
	30	93.81	30	95.10
Sodium chloride 9 *μ*g/mL	0	100.00	0	100.00
	30	89.99	30	98.93
Glucose 0.05 mg/mL	0	100.00	0	100.00
	30	94.34	30	95.97
Glucose 0.1 mg/mL	0	100.00	0	100.00
	30	98.10	30	94.32
Glucose 0.2 mg/mL	0	100.00	0	100.00
	30	94.89	30	99.06
Mixture of 9 *μ*g/mL sodium chloride and 0.05 mg/mL glucose (1 : 1 *V*/*V*)	0	100.00	0	100.00
	30	97.60	30	100.00
Mixture of 9 *μ*g/mL sodium chloride and 0.05 mg/mL glucose (1 : 2 *V*/*V*)	0	100.00	0	100.00
	30	97.99	30	92.89
Solutio Ringeri	0	100.00	0	100.00
	30	97.80	30	99.51
Solutio Ringeri Lactate	0	100.00	0	100.00
	30	96.76	30	100.00
Multielectrolytic isotonic fluid	0	100.00	0	100.00
	30	96.35	30	98.46
Pediatric fluid	0	100.00	0	100.00
	30	98.98	30	100.00

*c* (%): percent of initial concentration.
